# ASCO-update: gastrointestinal tumors

**DOI:** 10.1007/s12254-017-0366-9

**Published:** 2017-11-13

**Authors:** Lukas Weiss, Florian Huemer, Richard Greil

**Affiliations:** IIIrd Medical Department of Hematology, Medical Oncology, Hemostaseology, Rheumatology and Infectious Disease, Salzburg Cancer Research Institute (SCRI), Paracelsus Medical University, Muellner Hauptstraße 48, 5020 Salzburg, Austria

**Keywords:** Cololrectal cancer, Gastric cancer, Biliary tract cancer, Immune checkpoint inhibitor

## Abstract

A multitude of new molecular and immunologic insights into gastrointestinal tumors have been presented at the ASCO meeting 2017; however, we have focused our update on practice-changing phase 3 trials and tried to set them into clinical perspective. Furthermore, we will elaborate on updated data on immunotherapeutics in gastroesophageal cancers, since drug approvals may be anticipated before convening for the next meeting in 2018.

## Risk-adapted duration of adjuvant chemotherapy in stage III colon cancer

In stage III colon cancer, adjuvant chemotherapy using FOLFOX (5-Fluorouracil, Leucovorin, Oxaliplatin) or CAPOX (Capecitabine, Oxaliplatin) is well established due to its documented prolongation of disease free [1, 2] as well as overall survival [3].

However, oxaliplatin-induced peripheral neurotoxicity poses a relevant clinical problem affecting more than 90% of patients when treated with 6 months of FOLFOX and more than 10% with grade 3 or 4. Since oxaliplatin is associated with cumulative, dose-dependent toxicity, it is highly relevant to determine whether the duration of oxaliplatin-based chemotherapy (FOLFOX or CAPOX) can be shortened without losing clinical efficacy.

The IDEA (International Duration Evaluation of Adjuvant Chemotherapy) collaboration [4] pooled data from six phase III trials conducted in 12 countries and included more than 12,000 patients (see Table 1). These trials investigated the non-inferiority of 3 versus 6 months of adjuvant treatment with the primary endpoint of disease-free survival (DFS).

**Table 1:** IDEA trials

Trial	Regimen(s)	Stage	Tumor location	CAPOX (%)
TOSCA	CAPOX or FOLFOX4	II & III	Colon	35
SCOT	CAPOX or mFOLFOX6	II & III	Colon & rectum	67
IDEA France	CAPOX or mFOLFOX6	III	Colon	10
C80702	mFOLFOX6	III	Colon	0
HORG	CAPOX or FOLFOX4	II & III	Colon	58
ACHIEVE	CAPOX or mFOLFOX6	III	Colon	76

*FOLFOX* 5-Fluorouracil, Leucovorin, Oxaliplatin, *CAPOX* Capecitabine, Oxaliplatin

Patient characteristics were well balanced between the two treatment arms.

The estimated 3‑year DFS in patients receiving 3 months of adjuvant chemotherapy (74.6%) was lower than that in patients receiving 6 months of adjuvant chemotherapy (75.5%) by 0.9% (HR 1.07, 95% CI [1.00, 1.15]). Since the upper limit of the CI exceeded the predefined non-inferiority margin of 1.12 for the DFS hazard ratio, non-inferiority was not established.

Despite this formally negative trial, preplanned subgroup analyses have possibly led to practice changing results:

Patients with lower risk disease defined as T1–3 N1 represented roughly 60% of patients. In this subgroup, non-inferiority of 3 months therapy could be established when compared to 6 months therapy with a DFS HR of 1.01 (95% CI 0.90–1.12).

However, this was not the case for patients with higher risk disease defined as T4 or N2 (DFS HR 1.12, 95% CI 1.03–1.23), favoring 6 months over 3 months of adjuvant therapy.

In a not prespecified combined DFS comparison by risk group and regimen, true non-inferiority in lower risk patients was only found for CAPOX (DFS HR 0.85, 95% CI 0.71–1.01), but not for FOLFOX (DFS HR 1.10, 95% CI 0.96–1.26).

The investigators hypothesized that possible reasons for a superiority in CAPOX over FOLFOX may either lie in the higher oxaliplatin dose received in the first 4 weeks of treatment (CAPOX 260 mg/m^2^; FOLFOX 170 mg/m^2^) or in the more continuous exposure to capecitabine in the CAPOX regimen when compared to 5‑FU in the FOLFOX regimen.

The goal of lowering burdensome neurotoxicity by shortening treatment duration was achieved: patients receiving 3 months of treatment had significantly lower rates of grade ≥2 neurotoxicity (3 months vs 6 months: 17% vs 48% [FOLFOX] and 15% vs 45% [CAPOX]; *P* = 0.0001). Presumably as a consequence thereof, compliance was worse in the 6‑month arm than in the 3‑month arm, irrespective of the regimen used (3 months vs 6 months: 90% vs. 71% [FOLFOX]; 3 months vs 6 months: 86% vs 65% [CAPOX]).

### Take home message

Based on these results of the IDEA trials, the authors recommend a risk-adapted approach to selecting adjuvant chemotherapy duration for stage III colon cancer:

- T1–3, N1: 3 months of CAPOX or FOLFOX
- T4 or N2: 6 months of FOLFOX

Although patients with stage III rectal cancer were only included in the SCOT trial (without preoperative chemoradiotherapy), it might be appropriate to also extrapolate the above mentioned recommendations to rectal cancer.

## Adjuvant capecitabine for biliary tract cancers

The BILCAP trial examined adjuvant capecitabine in patients with resected biliary tract cancers [5]: in this two-arm, open-label, controlled study, 447 patients were randomly assigned to observation or to capecitabine (1250 mg/m^2^) twice daily for 8 cycles.

In the ITT population, capecitabine was associated with a median OS of 51.1 months (95% CI [34.6, 59.1]), compared to 36.4 months with observation (95% CI [29.7, 44.5]). Despite this 15-month difference statistical significance was not reached (HR 0.81, 95% CI [0.63, 1.04]; *p* = 0.097), which was however reached in the per-protocol population (HR 0.75, 95% CI [0.58, 0.97]; *p* = 0.028), with a median OS of 52.7 months in the capecitabine group compared to 36.1 months in the observation group.

Similarly significant improvements were also seen in relapse-free survival (RFS): 24.6 months in the capecitabine arm vs 17.6 months in the observation arm (HR 0.76, *P* = 0.039). Adverse events were well manageable with palmar–plantar erythrodysesthesia as the most common grade 3/4 adverse event (21%).

### Take home message

In the light of the significantly prolonged RFS, adjuvant capecitabine should become the standard of care in resected biliary tract cancers and should be used as control arm in future adjuvant trials. With great interest we are awaiting the results of the currently ongoing adjuvant ACTICCA-1 trial (NCT02170090) using gemcitabine and cisplatin which will challenge the role of adjuvant capecitabine.

## Perioperative FLOT for resectable gastric and GEJ cancer

The FLOT4 trial [6] compared perioperative chemotherapy with FLOT versus ECF/ECX in patients with resectable gastric cancer or adenocarcinoma of the gastroesophageal junction (types I to III; cT2–T4 or cT any and cN+). In this multicenter, randomized Phase 3 trial 716 patients were randomly assigned to receiving 4 preoperative and 4 postoperative cycles of FLOT every 2 weeks (docetaxel 50 mg/m^2^, day 1; 5‑FU 2600 mg/m^2^ over 24 h, day 1, leucovorin 200 mg/m^2^, day 1; oxaliplatin 85 mg/m^2^, day 1) versus receiving 3 preoperative and 3 postoperative cycles of ECF/ECX (epirubicin, cisplatin, 5‑FU or capecitabine).

The primary endpoint OS could be significantly improved with FLOT (median 50 months) when compared to ECF/ECX with a median OS of 35 months (HR 0.77, *P* = 0.012). Accordingly, PFS improved by 12 months in patients receiving FLOT (30 months vs 18 months; HR 0.75, *P* = 0.004). This effect could be consistently observed across histological and clinical subgroups.

Furthermore, FLOT resulted in a higher rate of pathologic remissions with 25% of ≤ypT1 versus 15% with ECF/ECX (*P* = 0.001).

Rates of serious adverse events were similar between the two treatment arms and perioperative FLOT did not increase surgical morbidity or mortality, the rate of resurgeries or hospitalization times when compared to ECF/ECX.

### Take home message

The final results of the FLOT4 phase 3 trial have been long awaited and now confirm the results of the previous phase 2 and a clinical practice which has already been adopted in many European centers. Therefore, FLOT can be considered the new standard of perioperative chemotherapy for resectable gastric or gastroesophageal adenocarcinoma.

In light of the fact that only half of the patients have completed their allocated postoperative chemotherapy, future clinical trials are aiming at the intensification of preoperative therapy or at employing new types of therapy in the postoperative setting such as immune checkpoint inhibitors.

## The role of immune checkpoint inhibitors in gastroesophageal cancers—updated and confirmed

Especially important from a European perspective: two trials mainly including patients from western countries have been updated at ASCO this year and could confirm the promising results observed in previous Asian trials:

The cohort 1 of the phase 2 trial KEYNOTE-059 has included 259 patients that have previously been treated with 2 or more lines of chemotherapy [7]. Patients were mainly recruited form the US (47.9%), only 13.1% from East Asia and 39.0% from the rest of the world.

PDL-1 expression was positive in 57.1% of patients (defined as a combined positive score of ≥1%) and although PD-L1 positivity was associated with a higher overall response rate (ORR) of 15.5%, PD-L1 negativity did not preclude patients from response (ORR 6.4%). Indeed, similar rates of complete responses were seen in the PD-L1 positive (2.0%) and the PD-L1 negative cohort (2.8%). However, a certain decline in ORR could be observed with an increasing number of previous lines of therapy (3 lines: 16.4%; 4 lines: 6.4%). Best responses were seen in patients with MSI high tumors, where a response was seen in 4 out of 7 patients.

Overall, treatment with pembrolizumab (200 mg every 3 weeks) resulted in an ORR of 11.6% and the reduction in tumor in 42.4% of patients.

The phase 1/2 CheckMate 032 trial compared the combination of nivolumab and ipilimumab to nivolumab monotherapy in 160 patients with advanced/metastatic gastric or gastroesophageal cancer [8].

Two different schedules of the combination therapy were compared to standard doses nivolumab (3 mg/kg IV every 2 weeks). Patients receiving the combination nivolumab (1 mg/kg) and ipilimumab (3 mg/kg) had an ORR of 24%, compared with 12% in patients receiving nivolumab alone. Of interest, ORR in the combinations arms seemed to be dependent on dosing, as only 8% patients who received the alternate dosing (nivolumab 3 mg/kg and ipilimumab 1 mg/kg) responded. Responses were observed regardless of PD-L1 expression.

As expected, combination therapy was associated with higher serious toxicity (NIVO 1 & IPI 3: 43%; NIVO: 10%).

### Take home message

The corroborated positive results obtained with immune checkpoint inhibitors in gastroesophageal cancers will pave the way for their implementation in the clinics following the anticipated approvals of pembrolizumab and nivolumab in 2018.
